# *“Peas in a pod”*: Oral History Reflections on Autistic Identity in Family and Community by Late-Diagnosed Adults

**DOI:** 10.1007/s10803-022-05667-z

**Published:** 2022-07-14

**Authors:** Rozanna Lilley, Wenn Lawson, Gabrielle Hall, Joanne Mahony, Hayley Clapham, Melanie Heyworth, Samuel Arnold, Julian Trollor, Michael Yudell, Elizabeth Pellicano

**Affiliations:** 1 Macquarie School of Education, Macquarie University, 29 Wally’s Walk, Sydney, NSW 2109 Australia; 2 Cooperative Research Centre for Living With Autism (Autism CRC), Brisbane, QLD Australia; 3 Independent autistic advisor, Sydney, NSW Australia; 4 Department of Developmental Disability Neuropsychiatry (3DN), School of Psychiatry, UNSW Sydney, Sydney, NSW Australia; 5 College of Health Solutions, Arizona State University, Phoenix, AZ USA; 6 Department of Clinical, Educational and Health Psychology, University College London, London, UK

**Keywords:** Autism, Late-diagnosed adults, Participatory research, Identity, Family, Community

## Abstract

In this paper, we report on a participatory oral history study documenting the lives of late-diagnosed autistic adults in Australia. We interviewed 26 autistic adults about their life history and the impact of late diagnosis. All were diagnosed after the age of 35, growing up in an era when autism was not well known. Using reflexive thematic analysis, we uncovered a rich body of reflections on shared Autistic identity and identified three major themes within that data set: ‘conceptualising the Autistic family’, ‘creating Autistic community’, and ‘contesting Autistic identity’. Overall, the study provides insights into the active creation of shared Autistic identity and the importance of Autistic community to these late-diagnosed autistic adults.

## Introduction

Since autism was first described as a clinical condition (Kanner, 1943), it has transformed from being conceptualised as a narrowly defined, rare disorder with childhood onset to a widely recognised and heterogeneous lifelong condition (Happé & Frith, 2020; Lord et al., 2018). The rise in autism prevalence, in part prompted by an expansion of the diagnostic criteria to include those without a clinically significant delay in language, cognitive development or adaptive behavior (American Psychiatric Association (APA), 1994), has led to an identification of previously undiagnosed adults as autistic,^1^ who were either misdiagnosed in childhood or not diagnosed at all. For this so-called “lost generation” of autistic adults (Lai & Baron-Cohen, 2015), a diagnosis of autism may result in profound changes not only to self-identity but also to their sense of collective identity as Autistic.^2^ The current study explores how these adults create a sense of shared or collective autistic identity with others, and the impact this had on their lives following their diagnosis.

Previous research has described adult autism diagnosis as a process of “biographical illumination” (Tan, 2018), potentially enriching self-identity and social relationships. Scholars of this process have noted that selves are relational achievements (Davidson & Henderson, 2010), in part because identity narratives are produced in and draw on interactions with others (Parsloe, 2015). Our study, co-produced by autistic and non-autistic researchers, adopts a relational framework to investigate perceptions of shared autistic identity in the everyday contexts of both family and community.

Overall, there is very little research investigating the formation of autistic identity within families or between family members, including autistic parents of autistic children. Kanner’s (1943) foundational description of autism as a “unique syndrome” specifically drew attention to potential trait similarities, such as “obsessiveness” and a preoccupation with abstractions, between parents and their autistic children. Since then, a large literature has emerged investigating the “Broader Autism Phenotype” (BAP), that is, the presence of autistic traits in undiagnosed family members of autistic individuals (e.g., Piven et al., 1997a), with attention focused on genetic mechanisms of transmission (e.g., Gerdts & Bernier, 2011; Sucksmith et al., 2011), especially in multi-incidence families (Bernier et al., 2012).

Much of this literature has highlighted negative aspects of familial patterns indicative of autism trait heritability, such as elevated rates of social difficulties (Bolton et al., 1994; Whitehouse et al., 2010) and psychiatric problems (Wolff et al., 1988; Yirmiya & Shaked, 2005) in both first- and second-degree relatives (Ingersoll & Wainer, 2014; Micali et al., 2004). An emphasis on the potential benefits rather than burdens of shared autistic identity within families has only recently emerged in academic literature. In 1988, Ritvo and colleagues first mooted the possibility that autistic children might mature into autistic adults who have their own children. In a later paper (Ritvo et al., 1994), they briefly considered the impact of autistic parenting on familial identity, noting that some parents described how they and their children were “so much alike” (p. 153) and felt that this similarity enhanced their mutual understanding.

A series of recent studies have further explored the potential for a positive sense of shared identity between autistic parents and autistic children. A phenomenological analysis of the experiences of autistic mothers found shared diagnoses helped participants to feel closer and more connected to their autistic children, encompassing an instinctive understanding of how best to meet their needs (Dugdale et al., 2021). Other studies have similarly reported that autistic parents stress the benefits of their own experiential expertise, including heightened empathy towards, and intuitive understanding of, their autistic children (Crane et al., 2021; Heyworth et al., 2022; Winnard et al., 2022). In particular, these parents are concerned to provide support for the ongoing development of a positive autistic identity anchored in the concept of neurodiversity for their children, celebrating autism as an inseparable aspect of self (Fletcher-Randle, 2022; Kapp et al., 2013). Together, these studies highlight ways in which autism diagnosis may contribute to a revised and positive familial autistic identity.

Moving beyond the intimacy of familial contexts, we find that numerous commentators have documented the creation of a shared Autistic identity amongst autistic adults. This identity is usually expressed as part of a joint commitment to self-advocacy situated within a neurodiversity framework (Bertilsdotter Rosqvist et al., 2013; Hurlbutt & Chalmers, 2002). Some of these studies have been authored by autistic scholars (e.g., Botha et al., 2022; Dekker, 1999; Singer, 1999) writing about “the importance of feeling a connection to other ‘like-minded’ people” (Milton & Sims, 2016, p. 529) preferentially fostered in autistic spaces (organised by autistic people for autistic people), which offer mutual acceptance and empathy (Bertilsdotter Rosqvist et al., 2015; Sinclair, 2010). The majority of these studies have focused on shared Autistic identity and friendships in online forums (Bargiela et al., 2016; Brownlow et al., 2015; Davidson, 2008; Parsloe, 2015), including blogs (Seidmann, 2020) and other social media (Mazurek, 2013). Some research has also pointed to the importance of face-to-face interactions between autistic adults as contributing to shared Autistic identity, sometimes described as Autistic community (Idriss, 2021). Botha and colleagues (2022) have recently reported on “autistic community connectedness”, comprising a sense of similarity between autistic people, valued autistic friendships and a shared commitment to political goals informed by neurodiverse understandings.

Such accounts are important because they move us away from longstanding stereotypes of “inborn disturbances of affective contact” (Kanner, 1943, p. 250), which have been highly influential in informing successive iterations of diagnostic criteria as well as the perspectives of autism researchers. As Jaswal and Akhtar (2019) pointed out, the idea that autistic people have atypical social behaviour is part of the current diagnostic criteria for autism (APA, 2013) with diminished social interest remaining central to social motivation accounts of autism (e.g. Abrams et al., 2013; Chevallier et al., 2012; see Clements et al., 2018). Focusing on shared autistic identity in family and community allows us to question these narrow stereotypes, adopting a more nuanced approach recognising heterogeneity in the social abilities of autistic people (Lasgaard et al., 2010; Sinclair, 2010) as well as the potential importance of shared autistic identity to wellbeing (Botha et al., 2022; Milton & Sims, 2016). Investigating the successes of autistic sociality (Ochs & Solomon, 2010), especially between autistic people (Botha et al., 2022; Milton, 2012), suggests that processes of shared identity formation may be pivotal to developing a positive sense of Autistic identity, connecting self-identity to group identity through the sharing of similar experiences and orientations that provide a feeling of belonging “to the same tribe” (Sinclair, 2010, p. 9) or “within neurotype ease” (Crompton et al., 2020, p. 1443).

The present study contributes to a small but gradually expanding body of qualitative research investigating the experiences and perceptions of late-diagnosed autistic adults. In a previous article (Lilley et al., 2021), we analysed perceptions of self-identity among the same group of late-diagnosed autistic adults, drawing on oral history interviews. In oral history, the aim is to record recollections, preserving voices and perspectives, often of those marginalised in conventional histories (Pellicano et al., 2020). The emphasis on the interviewees’^3^ ownership of their own story as well as the contextualisation of events and feelings in a broader arc of life experiences situated in the specificities of a particular time and place encourages rich and deep accounts. Such an approach, we suggest, elicits detailed reflections on self and others, ideal for investigating processes of identity formation. In the following we extend our previous research focus on self-identity by asking: How do late-diagnosed autistic adults create a sense of shared autistic identity with others, and what are some of the challenges to that process?

## Methods

To be included in this study, interviewees were required to (i) have been born before 1975, prior to the inclusion of autism in the Diagnostic and Statistical Manual – 3^rd^ edition (APA, 1980) and before there was much awareness of autism, (ii) have received an independent clinical diagnosis of an autism spectrum condition according to DSM-IV (APA, 2000) or DSM-5 (APA, 2013) in mid adulthood, after the age of 35 years; (iii) be English speaking; and (4) have spent most of their childhood and adulthood in Australia.

Twenty-six adults met these criteria, ranging in age from 45 to 72 years, with 14 identifying as female, 10 as male, one as non-binary and another “preferred not to say”. They had received their autism (n = 19) or Asperger’s (n = 7) diagnosis at, on average, 49 years of age. Some had received their diagnosis very recently (three months earlier), while others had received it 10 years prior to participation in this study (M = 3.14 years). All identified as white European ethnic background and one person also identified as Aboriginal. Most interviewees reported being well educated and currently employed (see Table 1). More than half (n = 14; 54%) reported having additional mental health and/or neurodevelopmental diagnoses, most commonly anxiety disorder (n = 12; 50%).

**Table 1:** Interviewee characteristics

Name/Pseudonym	Gender identity	Age (years) at interview	Highest education level	Number of children (autistic)	Diagnosis	Age at diagnosis	Time (years) since diagnosis
Anna	Female	56.7	Undergraduate degree	3 (2)	Autism	54	2.0
Annette	Female	50.9	Completed high school	3 (3)	Asperger’s	48	2.0
Cheryl	Female	59.2	Undergraduate degree	3 (0)	ASD	58	0.9
Craig	Male	55.7	College certificate	0	ASD	52	3.3
Danielle	Female	50.0	Graduate diploma	2 (0)	ASD	46	4.0
Lloyd	Male	51.1	Postgraduate degree	1 (1)	ASD	48	2.2
David	Male	49.9	Undergraduate degree	0	ASD	48	1.4
Dirk	Male	50.5	Undergraduate degree	2 (0)	ASD	48	0.8
Freya	Female	45.5	Undergraduate degree	1 (1)	ASD	42	3.2
Greg	Male	57.3	Undergraduate degree	2 (0)	ASD	56	0.6
Sarah	Female	47.9	Undergraduate degree	1 (1)	ASD	42	5.9
Janine	Female	54.9	Undergraduate degree	2 (2)	ASD	54	0.2
John	Male	50.8	Undergraduate degree	2 (2)	ASD	43	7.6
Veronica	Non-binary	72.4	Undergraduate degree	0	Asperger’s	62	10.2
Kristen	Female	46.3	Undergraduate degree	1 (1)	ASD	45	0.8
Lisa	Female	54.4	Undergraduate degree	2 (0)	ASD	51	2.0
Malcolm	Male	50.6	Undergraduate degree	0	Asperger’s	39	10.8
Peter	Male	69.6	College diploma	3 (0)	Asperger’s	68	1.2
Rebecca	Prefer not to say	45.3	Postgraduate degree	1 (1)	ASD	40	5.4
Scott	Female	52.8	Postgraduate degree	0	Asperger’s	45	5.8
Simon	Male	47.5	Undergraduate degree	0	ASD	45	2.8
Sarski	Female	46.0	Postgraduate degree	2	ASD	43	2.3
Tanya	Female	47.3	Postgraduate diploma	1 (1)	ASD	46	1.2
Wendy	Female	59.3	Postgraduate degree	2 (0)	Asperger’s	57	2.8
Jane	Female (cisgender)	46.9	Postgraduate degree	0	Asperger’s	43	2.0
Andrew	Male	56.5	Started high school	1 (0)	ASD	56	0.2
Mean (SD)		52.89 (6.83)				49.19 (7.19)	3.14 (2.87)

### Procedure

Ethical approval for this study was granted from Macquarie University’s Human Research Ethics Committee (reference no. 52019556310562). All interviewees provided written, informed consent prior to participation, and re-consented after having completed their oral history interview.

Interviewees took part in three separate sessions (see Pellicano et al., 2020, for full details including interview schedule). To begin, in *Session 1* (~ 60 min), interviewees met with an autistic interviewer (GH, JM; see below) to establish rapport, provide informed consent to participate in the study, and discuss the main interview, including the questions, and proactive wellbeing supports and strategies.

In *Session 2* (~ 1–3 h), interviewees completed the main oral history interview with the same autistic interviewer via their preferred means of communication, mostly Zoom (n = 23; 88%; two chose email, one face-to-face). Interviews ranged in duration from 41 to 189 min (M = 121 min). The interview proceeded chronologically through the different life stages, was semi-structured and sufficiently flexible to allow the interviewee to tell their life history in their own way.

In *Session 3* (~ 30 min), approximately one month later, interviewees reviewed their Session 2 interview transcript and re-consented for their materials to be included in the study. In the spirit of oral history methodology, most interviewees gave their consent for their full name (n = 15) or first name (n = 8) to be identified; five interviewees opted to use a pseudonym. Here, we use first names or pseudonyms, as preferred by our interviewees.

### Data Analysis

With the exception of the two email interviews, all interviews were recorded and professionally transcribed verbatim. We used reflexive thematic analysis (Braun & Clarke, 2006, 2019), including an inductive (bottom-up) approach (i.e., without integrating the themes within any pre-existing coding schemes or preconceptions of the researchers) to identify patterned meanings within the dataset specifically related to shared Autistic identity. The team brought a diverse range of perspectives to bear on the analysis, including psychology, anthropology, psychiatry, public health and history. Our epistemological stance fits within a critical realist framework, acknowledging the ways in which individuals make meaning of their experiences as well as the broader social context in which those meanings are constructed and used.

Members of the research team (GH, RL, WL, JM, & EP) met weekly for four months to reflect on, and discuss, each transcript, and consider codes and potential themes. RL then applied codes to all transcripts, drafting a thematic map of the potential themes and subthemes before sending it to the analysis team (GH, WL, JM, & EP) for comment and discussion. Team members liaised several times to review the thematic map and supporting quotations, focusing on semantic features of the data (staying close to participants’ language), resolving discrepancies, and deciding on the final themes and subthemes. Analysis was therefore iterative and reflexive (Braun & Clarke, 2006, 2019). Participants were also invited to review and comment on the manuscript and to exclude any quotes as part of a pre-publication member check.

### Community Involvement

The study involved three late-diagnosed autistic researchers and advocates (WL, GH & JM), who were actively involved as partners at every stage of the research process (Fletcher-Watson et al., 2019; Nicolaidis et al., 2011; Pellicano & Stears, 2011). Their involvement resulted in collaborative decisions that improved the relevance, clarity and accessibility of materials, and the nature and content of the oral history interview. All interviews were conducted by one of our autistic researchers (GH or JM), who were trained in oral history interview methods and supported by weekly team meetings.

The study was also overseen by an Autistic Advisory Group comprising three autistic adults (including MH & HC), who were reimbursed for their time and expertise, and provided detailed feedback on the participant information materials (including inclusion criteria) and data collection methods. Their input resulted in significant changes to all study information documents.

## Results

**Fig. 1:**
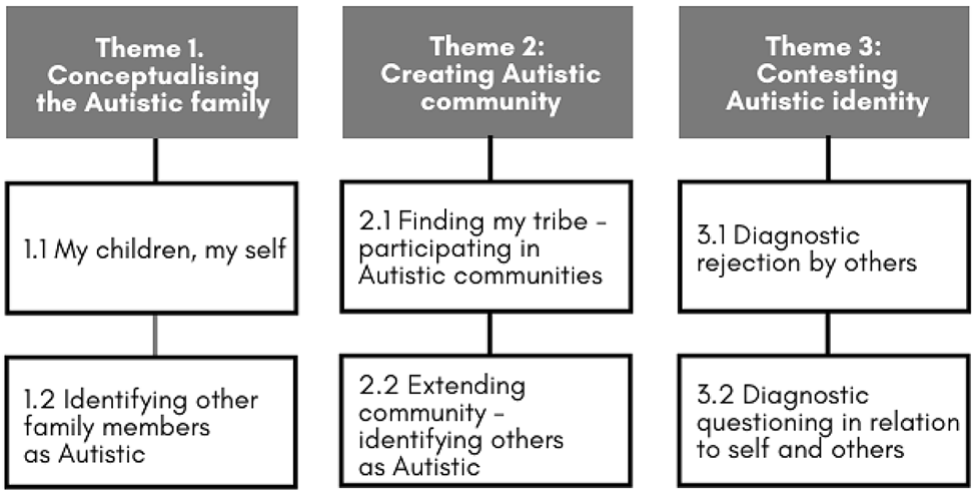
Themes and subthemes identified in our interviewees’ oral history reflections on shared Autistic identity

We identified three themes relating to shared Autistic identity (see Fig. 1). The first two, ‘Conceptualising the Autistic family’ and ‘Creating Autistic community’, refer to the ways in which interviewees construct a shared identity, building a sense of belonging. The third theme, ‘Contesting Autistic identity’, discusses disruptions to that shared identity in the form of diagnostic scepticism and rejection. Readers are advised that some of this material may be distressing with discussion of mental health issues, including negative responses to diagnostic disclosure and suicide.

### Theme 1: Conceptualising the Autistic Family

Interviewees often talked about their sense of identification with their autistic children and also identified other family members, immediate and extended, as autistic. Quotes in this section are anonymised to protect the identity of children and other family members.

#### Subtheme 1.1: My Children, My Self

Many of our interviewees reported that their autism diagnosis was precipitated by the diagnosis of their child or children. One mother of autistic teenagers said that her son’s diagnosis at age 8 was relatively straightforward but that she had to “*really push*” for her daughter’s diagnosis in high school. She saw her daughter as being similar in many respects to herself, leading her to believe that she, too, might be autistic: “*Now that I saw we’re like peas in a pod… I realised, okay, well I think that must be me too*”. Another mother explained that her son was diagnosed with autism aged two. About a year later, she sought a diagnosis for herself, which she described as her son’s “*gift*” to her, reflecting: “*So who knows, if my son hadn’t been born, maybe I never would’ve been diagnosed*”. Fathers, too, expressed these ideas of deep connection between parents and autistic children, often based on an idea of shared ways of thinking or “*wirings*”. In response to his wife’s concerns about their child, an interviewee said, “*he’s not different, he’s just a smaller version of me*”. Another interviewee explained that her own autism diagnosis followed her granddaughter’s diagnosis, emphasising “*how much like me she was just in so many ways and still is*”.

Interviewees sometimes stated that they sought an autism diagnosis for themselves to help their autistic children. “*I’m doing this for my kids*”, one mother commented in relation to her decision to seek a diagnosis. Another mother, who described her autistic son as “*the mirror you see later*”, saw her own autism diagnosis as important partly because it meant she could be an autistic role model to her son. She explicitly told him “*that his brain was like mine*” because “*we think really fast*” and spoke about the importance of teaching him that “*there’s nothing wrong with being different and this is just one way to be*”.

Interviewees also adopted a neurodiversity lens to describe the connections between themselves and their children with other neurodevelopmental diagnoses. For example, a mother reflecting on her daughter diagnosed with Rett syndrome stated:

We get thrown all these different variations because we need diversity, and she’s natural. She’s not unnatural. She’s not imperfect. So, she’s helped me to reflect back, too, I think. Because I have to stand up for her. And then I think, I should be standing up for me, too. I’m allowed to exist and I’m allowed to have a different point of view. I’ve got every right as everybody else to just exist, so she does as well.

Interviewees sometimes suggested that being autistic positively influences their parenting. One mother said that she wants “*to set an example for my son*” and that she hopes “*to give him the acceptance and support I never had*”. “*I was always forced to comply or do and be things I never wanted to do; I don’t do this with him*”, she explained. Another emphasised the value of her experiential expertise as an autistic person in parenting her autistic son “*in the way that I know that I would want to have been supported, in the way that I need to be supported, and, really, there’s not much else a psychiatrist could do for him, or a psychologist*”.

For others, their own diagnosis made them wonder if their child would also meet criteria for an autism diagnosis. For example, a mother remarked: “*My boy’s just like me; he’s not quite diagnosed yet, but he will be*”. Interviewees commented that if they had autistic children then “*there’s a good possibility you have autism yourself*” and, conversely, that if they are autistic, their children may also be.

#### Subtheme 1.2: Identifying Other Family Members as Autistic

Interviewees spoke about other members of their families, identifying them as either autistic or potentially autistic. Sometimes these family members, generally in younger generations (e.g., nieces and nephews), had received a clinical diagnosis. More frequently, interviewees explained that either their own diagnosis or the diagnosis of their child was a catalyst for their informal identification of other family members as potentially autistic. One mother said that after her son was diagnosed, aged 11, “*everything shifted for me*”. That shift involved not only identifying strongly with her son but also in identifying autistic traits in other members of her natal family, including her brother and her grandmother:

Oh my gosh, you’re like me. That’s why I understand you. Oh my gosh, you are exactly the same as my brother. Oh my gosh, Nan, the complete detachment. … That’s when I went, bingo, I know what’s going on. I know it comes from family.

Some interviewees reported that psychologists asked them to draw a family tree and discuss the characteristics of their family members. In the process, they identified autistic traits in a range of relatives: “*I’m sure if he* [father] *was around today he’d run through the tick boxes to be on the spectrum*”. One interviewee was very explicit about the ways in which his recent autism diagnosis had led him to reconceptualise the characteristics of natal family members: “*I’m starting to look at other people, not in an accusing way, but I’m starting to understand my mother. I’m starting to understand my son*”. Some recounted trying to persuade family members that they would meet criteria for an autism diagnosis – “*lots of women aren’t diagnosed and I think you’re autistic too, Grandma, and she’s just like ‘what?’*”. Others identified their spouses as autistic – “*I’m pretty sure he’s on the spectrum as well*” – or said that following their own diagnosis “*my husband ended up being diagnosed autistic*”.

Some interviewees spoke about a range of neurodevelopmental and mental health issues, including Attention Deficit/Hyperactivity Disorder and anxiety, in their families and saw autism as part of a broader genetic pattern. One woman, for example, attributed multiple deaths amongst her cousins to “*alcohol-related accidents*”, adding “*there was always something genetic going on with our family… I was sure there must be some sort of link between everything and it must be more than autism*”. Not being identified as autistic was seen as having a potentially tragic impact on mental health: “*My brother didn’t understand himself and took his life*”.

Family members identified as potentially autistic were spoken about in both positive and negative terms. One woman, for example, described her father as “*an extremely nasty man*”, who was very violent and “*narcissistic*”, reflecting “*I don’t know whether he was autistic – it’s possible*”. Generally, however, interviewees highlighted what they perceived to be positive characteristics they shared with their potentially autistic relatives: “*I’d like to think I’m very similar to my father – he has so much wisdom around him; definitely on the spectrum himself*”. In one case, an interviewee described his mother’s view that her daughter-in-law was “*a bit different*”. He explained: “*That’s because we’re all autistic, and she looks different because she’s neurotypical. She was the odd one out*”.

### Theme 2: Creating Autistic Community

Interviewees talked about the value of interacting with other autistic people in both face-to-face settings and online communities. They also spoke about their capacity to identify people to whom they are not related as autistic.

#### Subtheme 2.1: Finding My Tribe—Participating in Autistic Communities

Interviewees spoke about the importance of their friendships with other autistic people, describing these relationships as based on shared understandings and characteristics. Freya, for example, described her friendship with another late-diagnosed autistic mother of an autistic child: “*We’ve become, I suppose, closer and closer and mostly because I think when you’re autistic and you’re hyperverbal and you vent about stuff, you can do so in a different way than other people do*”. She also spoke about the support she receives from interacting on autistic women’s Facebook groups: “*Finding a tribe of other people who are as they are and can be proud of who they are has just made such a difference for me as a human being*”. For her, this connection with like others was linked with a confirmation “*of being completely normal*”, that is, of being like other autistic people – “*it validates my feelings and thoughts and memories for myself, of myself, in ways that I don’t otherwise get*”. Sarah described being “*part of a community now, people with autism and disabilities*”. She uses social media “*to connect with other autistic people*”, seeing this as an “*experience of sharing with peers*” that she did not previously have access to.

The sense of identity with other autistic people was often attributed to shared ways of thinking and perceiving. Sometimes intelligence was seen as the defining feature of these connections. Wendy, who identifies as “*an Aspie as opposed to autistic*” spoke about the importance of finding “*my own tribes… and that was the smart people groups*”, explaining “*I’ve always gravitated towards a certain type of person – we like talking about rocket science*”. John suggested that “*the autistic mind*” is very different from “*the neurotypical mind*”, and, like Dirk, cited Einstein, Isaac Newton, Michelangelo, Leonardo da Vinci and Galileo as examples. Peter attended an “*Aspie group*” and “*met a guy there who was just like me*”. “*It was uncanny*”, he reflected.

Others emphasised their connection to all autistic people. Greg, for example, described being autistic as “*an incredibly rich experience*” based on “*a completely different way of thinking*” that means “*we understand each other a lot better than the average person could understand us*”. Simon explicitly acknowledged the heterogeneity of the autistic spectrum, describing himself as “*an out and loud Aspie*” while having a sense of experiential connection with “*non-verbal autistic people*”, forged when he was a support worker: “*I fell in love with these people… And it was kind of like this is me but it’s not me*”. Lisa suggested some of the complexities of creating a viable self-identity in the period immediately following diagnosis: “*I sat there for a couple of days thinking, wow. And just repeating it in my mind, I’m autistic, I’m autistic, I’ve got Asperger’s, Asperger’s*”.

Tanya said that being part of “*a neurodiverse community*” gives her a feeling of having “*come home*”, of finding “*a place of acceptance*”. Jane referred to her online friendships with other autistic people as “*real friendships*” “*where you don’t have to pretend*”; Lisa said joining online groups “*and meeting yourself*” is “*empowering*”. Since her diagnosis, Anna only socialises with autistic people because “*these friendships are a completely different quality from others I’ve had in my life; there’s no need to be anything but ourselves*”. She referred to the effort of neurotypical interactions with “*all that fluffy stuff – the chit chat, the small talk*” and contrasted this with the joy of being able to speak for hours about interests with autistic friends. Kristen succinctly summarised the ways in which online communities can reinforce Autistic identity – “*the online community is fabulous for that because you read things and you’re like, that’s me and that’s me and that’s also me*”.

Some interviewees mentioned they would like to be involved in online Autistic communities but had not yet found a suitable group. Cheryl, for example, was interested in a local online group that offered information relevant to her area as well as the possibility of face-to-face interactions – “*I haven’t really found anything along those lines*”.

#### Subtheme 2.2: Extending Community—Identifying Others as Autistic

Interviewees sometimes mentioned that they are able to identify others as autistic who do not have a formal diagnosis and who may not identify as autistic. Jane said her friends are “*mostly on the spectrum but don’t know it*”. “*What I find is I can identify the autistic people in my life, but for the most part they either don’t know it or if they’re diagnosed they’re not talking about it*”, she elaborated.

Scott said she “*can quite often spot who the other autistics are*” at her university workplace, describing these colleagues as “*clearly autistic and not out*”. Kristen described one of her work colleagues in the following terms: “*And the thing is, while she’s undiagnosed, she’s autistic. Presents entirely differently to what I do, but she is*”. She added that they share “*hilarious quirks*” related to sensory hypersensitivities and rigidities, joking “*my way is the right way*”.

For some, the identification of autistic traits in others was a source of self-compassion and empathetic identification. Andrew mentioned that since his diagnosis he is “*seeing it in a lot of people*” and that this has given him “*better understanding and better tolerance of people who used to aggravate me*”. He spoke about his partner as having “*definite traits*” and suggested that her sense “*that she felt different*” was the basis of a deep connection between them – “*she just somehow instinctively saw that maybe I was someone who would accept her*”. Lloyd spoke about how he “*can now spot other fellow spectrumites and come from a position of compassion, because I’ve lived through what they are going to experience, which makes me cry for the pain, anxiety, and frustration they are probably going to go through*”. David, by way of contrast, said: “*I clash really strongly with people with traits that are very similar to mine. So my gut feel is that they’re undiagnosed*”.

Simon also referred to the role of intuition in identifying others as autistic, explicitly contrasting this experiential knowledge with professional expertise:

My mother is probably autistic, but again, it’s not for me to speculate… It’s just my intuition that’s saying that. I’m often right, but I know I can be wrong. I’m not a diagnostician. But autistic people, particularly people like me, with an interest in autism, can often have a good radar for that sort of thing.

### Theme 3: Contesting Autistic Identity

While interviewees often spoke about sharing a sense of Autistic identity with family members or the wider Autistic community, they also indicated that their diagnosis was not always accepted by others and that they sometimes questioned the Autistic identity of others and/or self.

#### Subtheme 3.1: Diagnostic Rejection by Others

A number of interviewees said that their autism diagnosis was questioned by their partner. In some cases this was because it is perceived as stigmatising. Craig, for example, said that his wife was worried that “*if people found out that I was autistic they would think of me as intellectually disabled*”. He suggested that “*saying your Asperger’s separates you*” from that stigmatised category. Another interviewee reported that his wife “*doesn’t believe it* [his autism diagnosis] *at all*”. Sarski explained that she is in the process of breaking up with her partner. She attributed the tensions in their relationship to his not accepting her autism diagnosis and, at the same time, not wanting to have a relationship with someone who is autistic. “*He wants a partner that’s neurotypical*”, she commented, adding:

It’s not something I can change about myself. I’m born this way. I’m proud of myself. I’m actually really a survivor in a way… We’ve been together for 25 years, so a label should be a key to understanding, rather than a reason to reject.

Interviewees reported that their parents sometimes rejected the validity of their autism diagnosis. Lisa said that, after she was diagnosed at 51, her father responded with, “*what a total load of rubbish*”. She described this diagnostic scepticism as “*quite hurtful*”, leading to a situation in which her natal family “*don’t talk about it – it’s like a big white bear in the corner of the room*”. Craig said that at first his natal family “*absolutely shot it* [his autism diagnosis] *down in flames as being completely wrong*”. “*If I had said, actually, I’ve discovered that I’m from Mars, it would have carried just as much weight*”, he conjectured. However, his mother became more accepting when she read the diagnosticians’ report, saying “*Oh, I can see a lot of your Dad in this explanation*”. Simon said that his mother’s rejection of his autism diagnosis is a source of conflict between them, attributing her reaction to a mix of denial and guilt that she did not support him better. Cheryl said that her mother “*was very dismissive*” of her autism diagnosis. She linked this reaction to the history of theories of autism causation, explaining:

She grew up in a time where they talked about the mother being to blame for children being autistic, and refrigerator mothers and all that sort of thing. So she was feeling like, if that was the case with me, then she might be partly to blame.

Cheryl said her autism diagnosis was also met with scepticism by her GP who told her, “*You seem perfectly fine to me*”. These doubts on the part of others, including family and professionals, made her wary of disclosing: “*So I haven’t told many people after that yet*”.

Other interviewees also spoke about the scepticism of health professionals. After her son was diagnosed, Kristen asked her GP for a referral to an autism diagnostician. When the GP asked her why she thought that applied to her, Kristen replied “*it’s a pervasive internal discomfort – I can’t explain it more than that*”. She said the GP noted on the referral that she made sufficient eye-contact and was neatly dressed. Danielle described wanting to be “*more open*” but feeling unsafe “*because I’m so used to it being contradicted or scoffed or people horrified at it*”. She cited the example of a psychiatrist who “*questioned my diagnosis*” and “*questioned why I would think that my son has autism*”. “*He just made me feel like an idiot, and I’m just not going back*”, she said.

#### Subtheme 3.2: Diagnostic Questioning of Self and Others

Some interviewees said that, at times, they question their own diagnostic status as autistic. Jane remarked: “*I wasn’t quite comfortable with disability either and a lot of signs were there that I was succeeding at things that ‘people like that’ don’t succeed at. So, could that be me?*” Sarski commented, “*maybe I’m not autistic enough*”, lamenting “*I’m so good at trying to be neurotypical that I’ve forgotten how to be autistic*”. She added, “*You get misunderstood a lot, even by your own community, if there is such a thing as your own community*”. Lisa described herself as a “*doubter*” with “*imposter syndrome*” who seeks reassurance from her counsellor that she is autistic – “*I thought I’m probably just making this up*”. Andrew explained that, in the past, “*I never thought of myself as autistic because I’m not that; I have a job, I have a wife, I can speak*”.

Peter was sometimes sceptical about other people’s claims to be autistic. Referring to a face-to-face Asperger support group he had attended, he observed “*I can see the little quirks in everybody but I think a lot of people were just in there to just be a bit of a social group*”. He even questioned whether the researcher he was speaking with was autistic: “*How are you autistic? You seem completely normal to me*”.

While these experiences of diagnostic rejection or self-doubt were common, interviewees also reported instances where others, both family and friends, did accept that they are autistic. Freya said she disclosed her autism diagnosis to her father who “*took it better than I expected*”. Sarski described the reaction of “*a long-term friend*” who responded to her disclosure in a very supportive way: “*He goes, ‘That makes so much sense; I want to know all about it; I want to know what this means’*”. Wendy, too, commented on a positive response to disclosure from “*close friends*” who said “*that doesn’t surprise me*”.

## Discussion

Taking an oral history approach, we recorded and thematically analysed the life stories of 26 autistic adults living in Australia, all diagnosed after age 35, verbally fluent and reported at least average intellectual functioning. Our study, coproduced by autistic and non-autistic researchers, highlights the active creation of shared Autistic identity, especially within families, and the importance of Autistic community to these late-diagnosed autistic adults.

The relationship between parent and child autism diagnoses has been mainly examined in the literature in terms of the presence of a BAP within families (e.g. Rubenstein & Chawla, 2018; Sasson et al., 2013). For example, elevated autistic traits in parents have been associated with greater parenting difficulties in relation to non-autistic children (Dissanayake et al., 2020). One Australian study of 22 adults (15 mothers and 7 fathers) and their children, all with a clinically confirmed diagnosis of Asperger syndrome, found reduced parental satisfaction among these adults compared to matched control parents of typically developing children (Lau & Peterson, 2011). In contrast, however, our interviewees described the development of a *positive* sense of shared Autistic identity with their autistic children. For some, this sense of deep connection was thought of as a “*gift*” that precipitated their own autism diagnosis relatively late in life. Recognition of shared autistic modes of being between (undiagnosed) parents and their autistic children was first suggested by Ritvo and colleagues (1994). Our findings echo this suggestion as well as more recent work documenting how a child’s autism diagnosis can prompt greater learning about autism and a process of comparison between self and child, which in turn can precipitate self-identification as autistic and formal adult autism diagnosis (Huang et al., 2021a; Powell & Acker, 2016; Ryan, 2013; Stagg & Belcher, 2019).

Our interviewees stated that they work hard to be positive Autistic role models and that they saw themselves as more efficacious parents because of their understanding and acceptance of their autistic children. This sense of intimate connection based on experiential knowledge, grounded in a recognition of the full personhood of their child (Lilley, 2011) and bonds of similarity that go “beyond the normal affinity of family” (Navon & Eyal, 2016, p. 1428), has been previously raised in relation to non-autistic parents of autistic children. While there is little literature on autistic parenting, more recent studies have highlighted autistic parents’ perception of a heightened empathy and understanding of their autistic children and their support for the ongoing development of a positive sense of Autistic identity grounded in valuing neurodiversity (Crane et al., 2021; Dugdale et al., 2021; Heyworth et al., 2022). Such findings provide empirical support for Milton’s (2012) model of the “double empathy problem” positing a disjuncture between autistic and non-autistic social actors leading to difficulties in social relationships. The parents in our study felt that being autistic allowed greater insight into the needs and disposition of their autistic children. It remains to be seen whether this relatively recent development of an explicitly conceptualised positive Autistic identity shared between parents and children positively impacts wellbeing over the longer term.

Researchers have described how genetic beliefs shape parent perceptions of autism causation and prognosis (Gray, 1994; Reiff et al., 2017) but the lay identification of diverse family members as autistic has not, to our knowledge, been previously discussed. Our interviewees identified their own siblings, parents, grandparents and/or spouses as (potentially) autistic. In so doing, they mobilised a primarily genetic understanding of autism, turning the diagnostic spotlight on both natal and affinal family members. Interviewees expressed a range of views – positive and negative – about their (potentially) autistic relatives, similar to existing research with autistic young people and adults (Cooper et al., 2021; Cribb et al., 2019; MacLeod et al., 2013; Mogensen & Mason, 2015). For some of our interviewees, seeing family members as possibly autistic provided an overarching explanation of mental health difficulties including tragic events such as suicide. It is important to note here that the self-knowledge of being autistic was presented as a protective factor in the context of mental health struggles and, conversely, not knowing one is autistic as a substantial risk factor. This position broadly supports research findings that autistic identity and greater personal autism acceptance can act as a protective factor against mental health difficulties (Cage et al., 2018; Cooper et al., 2017; Corden et al., 2021).

As Rapp and Ginsburg, (2011), writing about the broader context of disability narratives, have eloquently expressed it, “a diagnosis reverberates across a genealogy, revising understandings of the family tree” (p. 387). When autistic adults identify other family members, past and present, as (potentially) autistic or identify themselves as insightful autistic parents of valued autistic children they engage in a creative process of conceptualising the Autistic family. Most clinical constructs of the BAP focus on negative attributes using the language of “deficits” and “genetic liability” (e.g., Lainhart et al., 2002; Piven et al., 1997b), especially in multi-incidence families (Bernier et al., 2012). Our interviewees also highlighted the presence of autistic traits in multiple family members but they perceived the significance of this largely in terms of a recognition of a similar sensibility that formed part of their own active construction of a positively valued shared Autistic identity.

Our interviewees also highlighted how this shared sensibility extended beyond family members to other like-minded people within the Autistic community. The process of constructing Autistic community in online spaces was first noted by Blume (1997) and has been extensively documented as providing both a safe space for meeting other autistic adults (Seidmann, 2020), developing friendships (Bargiela et al., 2016; Brownlow et al., 2015) and for claiming a positive autistic identity, centred on being different not deficient, often linked to self-advocacy movements (Brownlow & O’Dell, 2002; Parsloe, 2015). Foundational Autistic activist Jim Sinclair (2010) described the importance of both the internet and face-to-face interactions in forging “shared autistic space” in which people have a sense of “belonging to the same tribe” as they form connections and create community. More recently autistic community has been conceptualised as grounded in “within-neurotype ease” (Crompton et al., 2020) or “belongingness” allowing a sense of instant connection encouraging the expression of their authentic autistic selves.

Interviewees stressed the importance of both face-to-face and online interactions in building friendships and peer support networks with other autistic adults, sometimes linking this to a discourse of autistic normalcy (being similar to other autistic adults) (Bertilsdotter Rosqvist, 2012; Hickey et al., 2018). Crompton and colleagues (2022, p. 9) have also noted the “relational and emotional benefits of autistic-autistic interaction” in a recent study of post-diagnostic peer support in adulthood. Interactions with other autistic adults provided our interviewees with self-validation through comparison with like others (“*that’s me and that’s also me*”), sometimes referred to using the metaphor of belonging to a (neuro)“*tribe*” (see Silberman, 2015). For some, that sense of shared community was part of a continuing identification with Asperger’s, which may still function as an identity grouping even though it is no longer a diagnostic label (Botha et al., 2022; Giles, 2014). Others were more inclusive in their orientation, explicitly acknowledging the heterogeneity of the autism spectrum and a common identity for everyone diagnosed autistic based on shared ways of thinking (“*the autistic mind*”) and perceiving the world. Learning to advocate for Autistic rights via online forums was explicitly mentioned as a vital part of online community (Bagatell, 2010; Dekker, 1999).

Interviewees sometimes suggested that they had a “*good radar*” for identifying undiagnosed others as autistic. In doing so, they emphasised the value of their experience-based knowledge of autism (Pellicano et al., 2019). For most, the identification of others as autistic was perceived in positive terms and as a basis for friendship or romantic attachment. This informal lay diagnostic practice extends the potential membership of Autistic community, building a sense of connection with significant others based on empathetic recognition. This has implications for the idea that social motivation deficits are central to understanding autism (Chevallier et al, 2012). A desire to identify others as autistic, thereby enlarging Autistic community, suggests that autistic adults may sometimes be preferentially socially motivated to engage with other autistic people, whether as family members, friends or sexual partners.

Interviewees constructed a viable sense of Autistic identity through conceptualising the Autistic family and creating Autistic community. In so doing, they found a sense of belonging and extended that sense of connection to others, including family members and consociates, either diagnosed or identified as autistic, sometimes conceptualised as extended family or fictive kin (“*my tribe*”). These practices both acknowledge pre-existing ties (such as parent–child relationships) and create new bonds of social connection with others (such as members of Autistic online communities).

Nevertheless, these active creations of social connections based on Autistic identity were not always successful or trouble free. Some of our interviewees reported that their late-in-life autism diagnosis was met by scepticism or rejection or that they sometimes questioned their own diagnostic status or the claims of others to be autistic. Autistic identity may be contested even when it is based on a formal clinical diagnosis. Interviewees suggested a number of reasons others might reject their diagnosis including i) the perception of autism as a stigmatising category (especially compared to Asperger’s disorder, removed from the DSM in 2013 (APA, 2013; see Giles, 2014)), ii) their ability to successfully “mask” or “camouflage” their autistic traits (see Cook et al., 2021; Hull et al., 2017; Lai et al., 2017; Lawson, 2020) and iii) their normative life trajectories and achievements such as marriage, raising children, postgraduate study or employment (see Atherton et al., 2021; Huang et al, 2021a). In these ways, our late-diagnosed interviewees felt they did not always match people’s pre-existing stereotypes of what an autistic person is capable of or behaves like (Leedham et al., 2020). Diagnostic scepticism or rejection by family members and partners may be partially motivated by an effort to avoid affiliate, or courtesy, stigma (Turnock et al., 2022). It may also be that a late diagnosis of autism substantially disrupts the ways in which consociates have previously thought about, and related to, an individual, making them resistant to reframing their perspective on that person. Scepticism about autistic individuals being “really” autistic, often based on a view that autism is overdiagnosed (Kapp & Ne’eman, 2020), has been widely reported in the literature, especially in relation to girls and women (Anderson, 2020; Bargiela et al., 2016; Bertilsdotter Rosqvist, 2012; Milner, 2019) and late-diagnosed adults (Crane et al., 2018; Finch et al., 2022; Huang et al., 2021b, 2022; Lewis, 2016). Diagnostic scepticism from healthcare providers about adult autism diagnosis has also been previously noted (Bargiela et al, 2016; Finch et al., 2022; Huang et al., 2021b; Jones et al, 2014) as well as autistic females being underserved by clinical criteria and diagnostic processes (Estrin et al., 2020).

Diagnostic questioning of self as autistic has been conceptualised as part of a “reflexive project of the self” (Giddens, 1992), involving “reflection, doubt, evaluation and uncertainty” (Bertilsdotter Rosqvist, 2012, p. 124). One of our interviewees described this diagnostic doubt as a form of autistic “*imposter syndrome*”. This diagnostic self-doubt has received limited analytic attention. Punshon and colleagues (2009, p. 274) reported that one of their late-diagnosed participants felt constantly anxious that his Asperger’s diagnosis would be revoked while Autistic activist Jim Sinclair (2010) noted that autistic heterogeneity can lead some autistic people to question their own or other people’s status as autistic. While autistic people may often reject self-understandings framed within a model of pathology (Botha et al., 2020), some of our interviewees suggested that negative framings and perceptions of autism can make them doubt their own diagnostic status as well as that of others as either too successful or too “normal” to be autistic. This internalisation of stigma sometimes coexisted with a rejection of stigmatising attitudes towards autism as individuals navigated complex feelings and reactions to late-diagnosis.

Given findings that a stronger autistic identity is related to positive self-esteem (Cooper et al., 2021), reports of both diagnostic rejection by others and diagnostic questioning in relation to self and others are concerning. Certainly, our interviewees expressed considerable distress at diagnostic rejection, especially by family members and partners. Previous research has noted the complex emotional reactions of parents (Legg et al., 2022; Raymond-Barker et al., 2018) and of non-autistic partners in neuro-mixed couples to adult autism diagnosis (Lewis, 2017a) but has not discussed instances of diagnostic rejection experienced by late-diagnosed autistic adults.

Our study contributes to the emerging literature on the complexities of autistic sociality (Ochs & Solomon, 2010), based on strong feelings of social identification with other autistic people (Maitland et al., 2021), and within-neurotype ease (Crompton et al., 2020), also described as “neurodivergent intersubjectivity” (Heasman & Gillespie, 2019). Our findings suggest that late-diagnosed Autistic identity narratives are inherently social, being produced through interaction with and reference to others, drawing on discourses that frame autism as a shared way of being in the world and that explicitly value Autistic community. Previously we have argued that, contra research suggesting autistic impairments in self-awareness, our late-diagnosed interviewees demonstrate a deep capacity for self-reflection (Lilley et al., 2021). The current analysis strengthens and extends this argument. Our interviewees highlighted the importance of shared Autistic social identity in family and community and their active role in shaping that shared identity. While these processes of shared identity building may show some similarities with the identity-based politics of other marginalised groups (Davidson & Henderson, 2010), an important difference is that, historically, autism has been thought of as primarily a social disorder, and this entrenched paradigm continues to inform clinical and scholarly views (Jaswal & Akhtar, 2019). Current diagnostic criteria for autism specify persistent deficits in social interaction exemplified by deficits in developing and maintaining relationships (APA, 2013; World Health Organization, 2019). In stark contrast to these views, our late-diagnosed interviewees, by engaging in kin- and community-based Autistic identity building, demonstrated both marked social identification with and social motivation towards others who are autistic or believed to be so. This suggests a potential disconnect between the experiences of at least some autistic adults and current diagnostic criteria relating to impairments in social interaction (see also Pellicano et al., 2021).

## Limitations and Future Directions

The late-diagnosed interviewees who generously shared their life stories for this study generally embraced their Autistic identity and saw themselves as part of a broader Autistic community. They were motivated to participate in this oral history study and felt competent to share aspects of their autobiographical journeys, in part because they were intellectually able and verbally articulate. Many explicitly aligned themselves with a neurodiversity approach. Their views may not apply to all late-diagnosed autistic adults or autistic people. Neither thematic analysis nor oral history claim to be representative or generalisable (Braun & Clarke, 2019). Rather, these qualitative methods provide nuanced accounts of particular people in a particular place and time. Given the heterogeneity of autism, and the great diversity of lived autistic experience (Dinishak, 2021), that particularity is appropriate to the study of autistic lives.

This Australian project opens up many areas for future research, including the extent to which late-diagnosed autistic adults in other cultural contexts actively build a shared Autistic identity based in family and community and whether or not that identity is, at times, contested. Given the growing phenomenon of self-diagnosis (Sarrett, 2016), in part prompted by numerous barriers to formal adult autism diagnosis, both within Australia (de Broize et al., 2021) and elsewhere (Lewis, 2017b), further research with these adults may illuminate aspects of the active creation of neurocommunities in the absence of clinical identification. Our focus on the processes of shared Autistic identity building highlights the value of participatory approaches, co-designed and co-produced by autistic and non-autistic researchers (see den Houting et al., 2021; Pellicano & den Houting, 2021), that privilege autistic perspectives and experience. Such approaches, we suggest, enable a move away from deficit-focused accounts of impaired autistic sociality towards a recognition of autistic adults as active agents creating a unique sense of family and community grounded in a positively conceptualised shared Autistic identity.

## Footnotes

1 Throughout we use ‘identify-first’ language (‘autistic person’), rather than person-first language (‘person with autism’), because it is the preferred term of our autistic co-researchers and of the people we interviewed (see Bury et al., 2020; Kenny et al., 2016) and is less associated with stigma (Gernsbacher, 2017).

2 We sometimes use autistic with a capital A to indicate interviewees’ identification with a shared community based on a common acceptance of a neurodiversity framework and a commitment to advocating for Autistic acceptance and social justice. This usage is adopted by Autistic activists and scholars (e.g., Benham & Kizer, 2016) who adopt this tactic of capitalisation from Deaf activists (Friedner & Block, 2017) to indicate that they are members of the Deaf community and regard themselves as culturally Deaf as distinct from “deaf” people who adopt a medical model of hearing impairment (Napier, 2002).

3 We use ‘interviewees’ rather than ‘participants’ throughout this paper, as this is accepted usage in oral history (Oral History Association, 2009).
